# Sketches and Illustrations of Medical Quackery—Homœopathy

**Published:** 1848-01

**Authors:** 


*Sketches and Illustrations of Medical Quackery.—Homœopathy.* The following case of administering powerful drugs in large doses under the guise of homœopathy, is noticed in the Medical Gazette, as having recently occurred in London:

"A lady who had been attended by a highly respectable general practitioner, recently consulted a homœopathic physician, who has acquired some celebrity in the fashionable quarter of the metropolis, for his skill in treating and *curing* diseases by infinitely small doses. She received from him four small white powders, with explicit directions, (now lying before us), one to be taken every other night,—each powder being numbered, and the night on which it was to be taken, as well as the mode of taking it, being particularly specified—'all dry on the tongue.' No. 1 was swallowed according to order, and the patient was soon afterwards seized with great sleepiness, stupor, and other alarming symptoms indicative of the action of a powerful narcotic. These effects were followed by diarrhœa. The patient was alarmed, and instead of looking upon the result as an indication of the beneficial working of homœopathic powders, or as a means of curing her of any latent skepticism respecting the efficacy of infinitely small doses, she was prudent enough to return to her old medical friend, to whom she handed the remaining powders, with the directions. This gentleman, suspecting that they contained some active narcotic, caused them to be submitted to a chemical analysis. We have now the report of this analysis before us, and of it we shall make the following abridgment. The powders were numbered 2, 3, and 4. They were *similar in appearance*, except that No. 3 was somewhat whiter than the other two: there was nothing to indicate that they were of different composition; and as they were to be taken in the same way on alternate nights, this could not possibly be suspected.

"Although there was no great dissimilarity in bulk, the powders were very unequal in weight. No. 2 weighed 3-4 grains; No. 3, 1-5 grains; No. 4, 2 grains. No. 2 was found, upon analysis, to consist entirely of calomel and *morphia*, the morphia forming not less than *one grain.* No. 3 contained no morphia or calomel. nor any mineral or other substance, but merely *sugar of milk.* No. 4 was composed of calomel and morphia, the morphia amounting to one half grain."

*Pro. Med. and Surg. Jour.*

